# Supplementary material for: Optimal enzyme utilization suggests that concentrations and thermodynamics determine binding mechanisms and enzyme saturations
Source: Nat Commun. 2023 May 5;14:2618. doi: 10.1038/s41467-023-38159-4 (PMC10162984; doi:10.1038/s41467-023-38159-4)
Supplement: Supplementary file 1 — Supplementary Information [file 41467_2023_38159_MOESM1_ESM.pdf]

Optimal enzyme utilization suggests that concentrations and thermodynamics determine binding mechanisms and enzyme saturations

Asli Sahin[a], Daniel R. Weilandt[a,b], Vassily Hatzimanikatis [a,*]

[a]*Laboratory of Computational Systems Biotechnology, Ecole Polytechnique Federale de Lausanne (EPFL), 1015 Lausanne, Switzerland*

[b] Present address: *Department of Chemistry and Lewis-Sigler Institute for Integrative Genomics, Princeton University, Princeton, New Jersey, United States*

*vassily.hatzimanikatis@epfl.ch

Supplementary Information

Supplementary Note

Discussion and comparison of the existing mathematical methodologies on enzyme optimality

In this section, we provide a general discussion and comparison of the methodologies used in the context of enzyme optimality. Numerous optimization methods have been previously discussed in the literature regarding enzyme optimality. These approaches differ based on the assumptions made, mechanistic details included, and evolutionary objectives studied. Therefore, they employ different classes of optimization methods to address the problem. We can group these methods into two parts based on the system variables studied in the context of enzyme optimality. The first part of the methods focuses on metabolite and enzyme levels as system variables, whereas the second part of the methods focuses on enzyme kinetic parameters as system variables.

The first part of these methods uses decomposed rate laws and includes kinetic parameters as input to the optimization problem and parameterizes the problem based on the measured or average kinetic data[1–4]. They focus instead on the metabolite and enzyme concentrations as system variables. Given a pathway flux profile, these methods estimate the metabolite and enzyme levels that minimize the cost of the enzyme while ensuring thermodynamic feasibility. Depending on the enzyme cost function and the search space, the emerging problem can be convex-linear[3], convex-non-linear[1], or non-convex[2]. While convex problems are easily solvable with traditional solvers, non-convex problems might become computationally intractable for large-scale nonlinear problems. Another approach assumes reactions to follow irreversible Michaelis-Menten kinetics and derives the

optimality condition between the concentrations of the substrates and the enzymes[4]. In all these methodologies, kinetic parameters describing the catalytic efficiency of an enzyme are treated as parameters, with no mechanistic or theoretical explanation for the assumed values.

The second part of these methods considers enzyme kinetic parameters as system variables. In these studies, kinetic properties, usually along with the enzyme concentrations, are treated as variables to the optimization problem[5–9]. These methods aim to understand how evolutionary and biophysical pressures shape the catalytic properties of cellular enzymes. Most of these methods employ population-based metaheuristic optimization methods[7–9], where a random mutation is inserted, and its fitness effect is evaluated at each iteration. This class of optimization methods require configuring several parameters to achieve the best performance with no guarantee on global optimality or convergence. They mechanistically link the enzyme's catalytic efficiency to a defined fitness function and study the fitness landscape of the enzymes without pushing the kinetic parameters to their biophysical limit[7–9]. However, they mainly focus on the evolution of enzyme turnover numbers (k_{cat}'s) and its effects on network topology[7]. They do not account for the reaction kinetics in detail with detailed saturation and thermodynamic terms[7,8], or they mainly focus on Michaelis-Menten kinetics[9].

Apart from the recent metaheuristic approaches, Heinrich and coworkers also analyzed the optimization of the kinetic parameters of enzymes by solving a nonlinear optimization problem [5,6]. Their method relied on an initial step where they first derived all possible types of optimal solutions with respect to the elementary rate constants assuming non-maximal values. After this step, they solved the optimization problem using Lagrange multipliers for each optimal solution. Although their method provides a detailed mechanistic understanding of the evolutionary pressure that shapes the catalytic optimality of enzymes, it addressed the nonlinearity of the problem by solving it locally for each optimal solution derived in the initial step and was generalized only for the ordered enzyme mechanisms.

Based on the system variables considered, our framework takes place in the second part of the methodologies, where the enzyme's catalytic efficiency is regarded as the evolutionary target. However, the mechanistic details and optimization methods utilized widely differ from the methods mentioned above. Firstly, our framework models reaction kinetics at the elementary reaction level and explicitly accounts for the enzyme saturation and thermodynamic constraints. Therefore, our framework can be applied to study any reaction mechanism and model enzyme activation and inhibition mechanisms. Although existing approaches have proven useful to model various enzyme mechanisms, completely randomized multi-substrate reaction mechanisms produce extremely complex rate equations[10], and modeling these reactions may be very difficult or even impossible with the existing methodologies. Our framework provides a mechanistic understanding of the selective pressures that shape the catalytic optimality of these complex mechanisms. Moreover, the parametric domain of our framework can be mapped for a broad range of metabolite concentrations and estimate context-specific theoretical upper bounds for the catalytic efficiency of enzymes.

Unlike the existing metaheuristic approaches that require tuning many parameters, our method addresses the problem in a similar fashion to Heinrich and coworkers [5,6]. Like the previous work, we constrained the elementary rate constants by their biophysical limit and utilized normalized concentrations and variables. However, to solve the resulting nonlinear problem more efficiently and to ensure global optimality, we addressed the nonlinearities and recast the problem as a MILP problem, which can be solved by traditional MILP solvers. The key point in this formulation is the discretization and the linearization of the bilinear products. By approximating elementary thermodynamic displacements with a piecewise constant function, we can solve the problem efficiently and capture the results previously obtained by analytical formulation. While the analytical formulation can give the exact solution for ordered enzyme mechanisms, our formulation includes an approximation; however, with correct binning and without a high cost in computational time, we can control the accuracy of the solutions to the decimals and obtain optimal modes of operations for any enzyme mechanism.

Reversible Michaelis-Menten Mechanism

As described in the main text, our formulation captures different kinetic designs at a catalytically optimal state depending on the reactant concentrations for the three-step reversible Michaelis-Menten mechanism. Our results show the sensitivity of the net steady-state flux over the concentration space and subdivision of the concentration space into different regions with distinct binding characteristics (Supplementary Fig. 1). The regions are defined with respect to the elementary rate constants assuming their submaximal values (See Supplementary Table 1). The results presented are in accordance with previous theoretical studies[6,11].

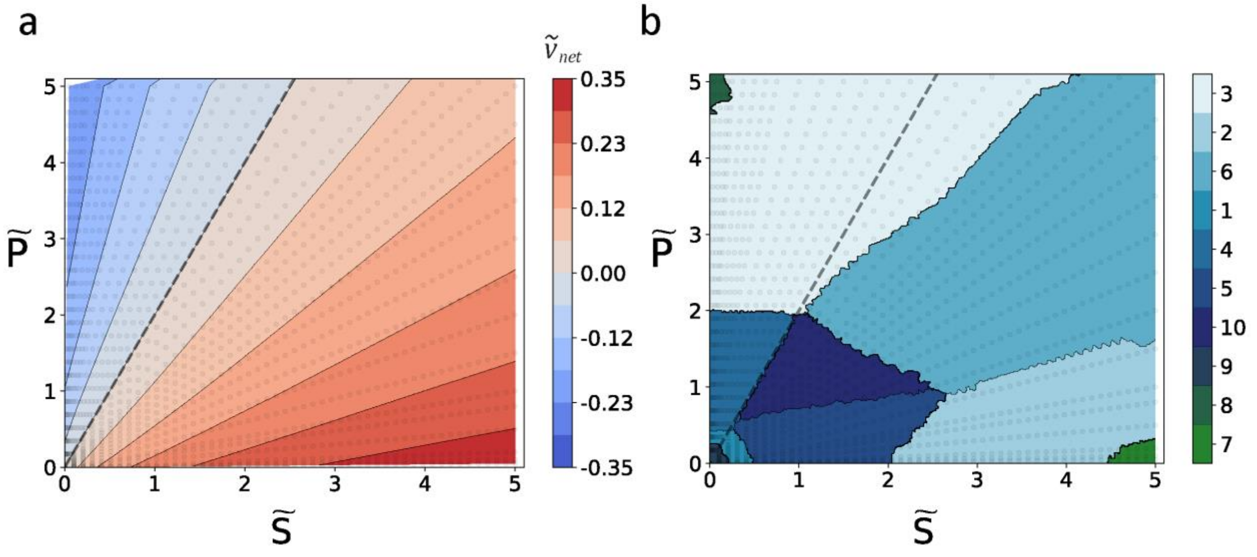

Supplementary Fig. 1: The net steady-state flux ($\tilde{v}_{net}$) and the regions of different kinetic designs at the optimal state for the reversible Michaelis-Menten mechanism (reproduced from previous theoretical studies[6,11]). a) Contour plot of the net steady-state flux ($\tilde{v}_{net}$) at optimal state for the

reversible Michaelis-Menten mechanism for $\widetilde{K}_{eq} = 2$, scatter points represent sampled points b) Contour plot for the regions based on different kinetic designs (defined with respect to the elementary rate constants at their submaximal values See Supplementary Table 1) reproduction of the regions from previous theoretical studies, colours indicate labels for different kinetic designs, numerated according to their original derivation[6,11]. The boundaries of regions are generated using the k-nearest neighbor classifier (k=13). Data is shown for $\widetilde{K}_{eq} = 2.0$. Dashed line represents the equilibrium line where $\tilde{P} = \widetilde{K}_{eq}\tilde{S}$. Source data are provided as a Source Data file.

Supplementary Table 1: Optimal solution types for the elementary rate constants of the three-step reversible Michaelis-Menten mechanism. Indicated rate constants take submaximal values for the given kinetic design ($k_{i,b,f} < 1$), whereas the remaining rate constants are at their maximal values ($k_{i,b,f} = 1$) (See Supplementary Fig. 1 b)

Label	Submaximal rate constants
1	$k_{1,b}$
2	$k_{2,b}$
3	$k_{3,b}$
4	$k_{1,b}, k_{3,b}$
5	$k_{1,b}, k_{2,b}$
6	$k_{2,b}, k_{3,b}$
7	$k_{1,f}, k_{2,b}$
8	$k_{2,f}, k_{3,b}$
9	$k_{3,f}, k_{1,b}$
10	$k_{1,b}, k_{2,b}, k_{3,b}$

Calculation of macroscopic kinetic parameters

Our formulation addresses the problem at the elementary reaction level and estimates elementary rate constants at a catalytically optimal state for the given operating conditions. The elementary rate constants can be translated into macroscopic kinetic parameters e.g., $k_{cat,f}, k_{cat,b}, K_{M,S}$ and $K_{M,P}$ with the following equations for the three-step reversible Michaelis-Menten mechanism shown in Eq. (1) in main text.

$$k_{cat,f} = \frac{k_{2,f}k_{3,f}}{k_{2,f} + k_{3,f} + k_{2,b}} \tag{1}$$

$$k_{cat,b} = \frac{k_{1,b}k_{2,b}}{k_{2,f} + k_{1,b} + k_{2,b}} \tag{2}$$

$$K_{M,S} = \frac{k_{2,f}k_{3,f} + k_{1,b}k_{3,f} + k_{1,b}k_{2,b}}{k_{1,f}(k_{2,f} + k_{3,f} + k_{2,b})} \tag{3}$$

$$K_{M,P} = \frac{k_{2,f}k_{3,f} + k_{1,b}k_{3,f} + k_{1,b}k_{2,b}}{k_{3,b}(k_{2,f} + k_{1,b} + k_{2,b})} \tag{4}$$

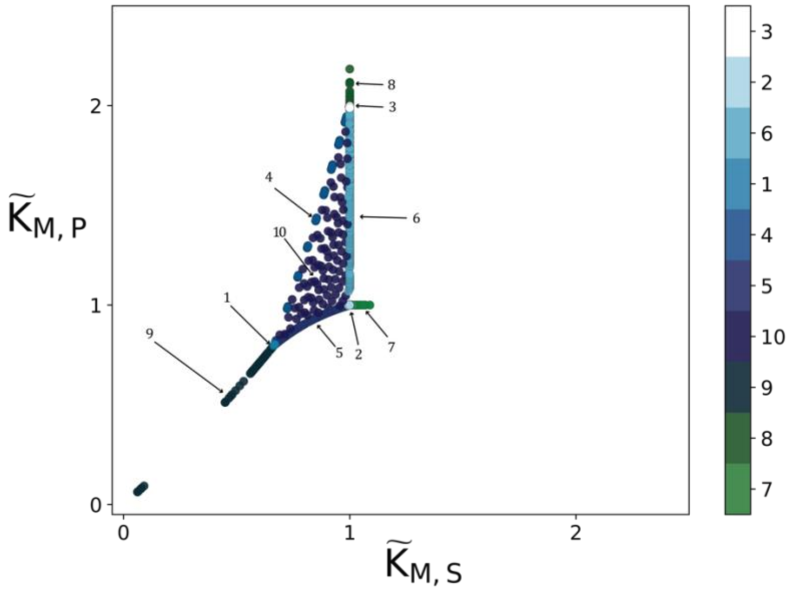

Supplementary Fig. 2: Michaelis-Menten constants $K_{M,S}$ and $K_{M,P}$ of the optimal states for the reversible Michaelis-Menten mechanism, for $\widetilde{K}_{eq} = 2$, colors indicate the kinetic designs at optimal states shown in Supplementary Fig. 1 b and in Supplementary Table 1. The results are in accordance with the previous studies[6,11]. Source data are provided as a Source Data file.

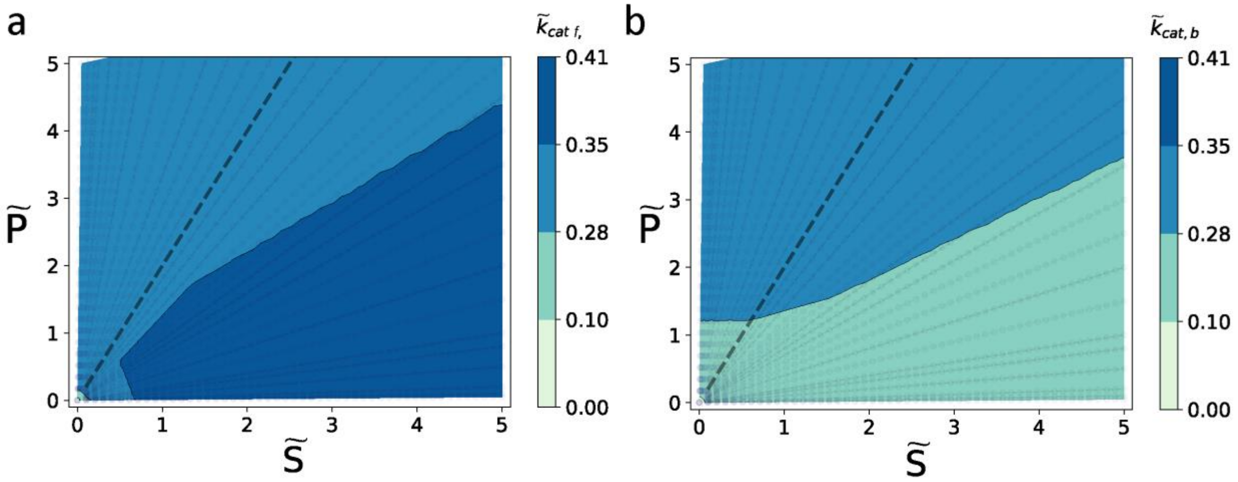

Supplementary Fig. 3: Turnover numbers, a) $k_{cat,f}$ and b) $k_{cat,b}$ in optimal states for the reversible Michaelis-Menten mechanism for $\widetilde{K}_{eq} = 2$, colours indicate the value of k_{cat}. Dashed line represents the equilibrium line where $\tilde{P} = \widetilde{K}_{eq}\tilde{S}$. Source data are provided as a Source Data file.

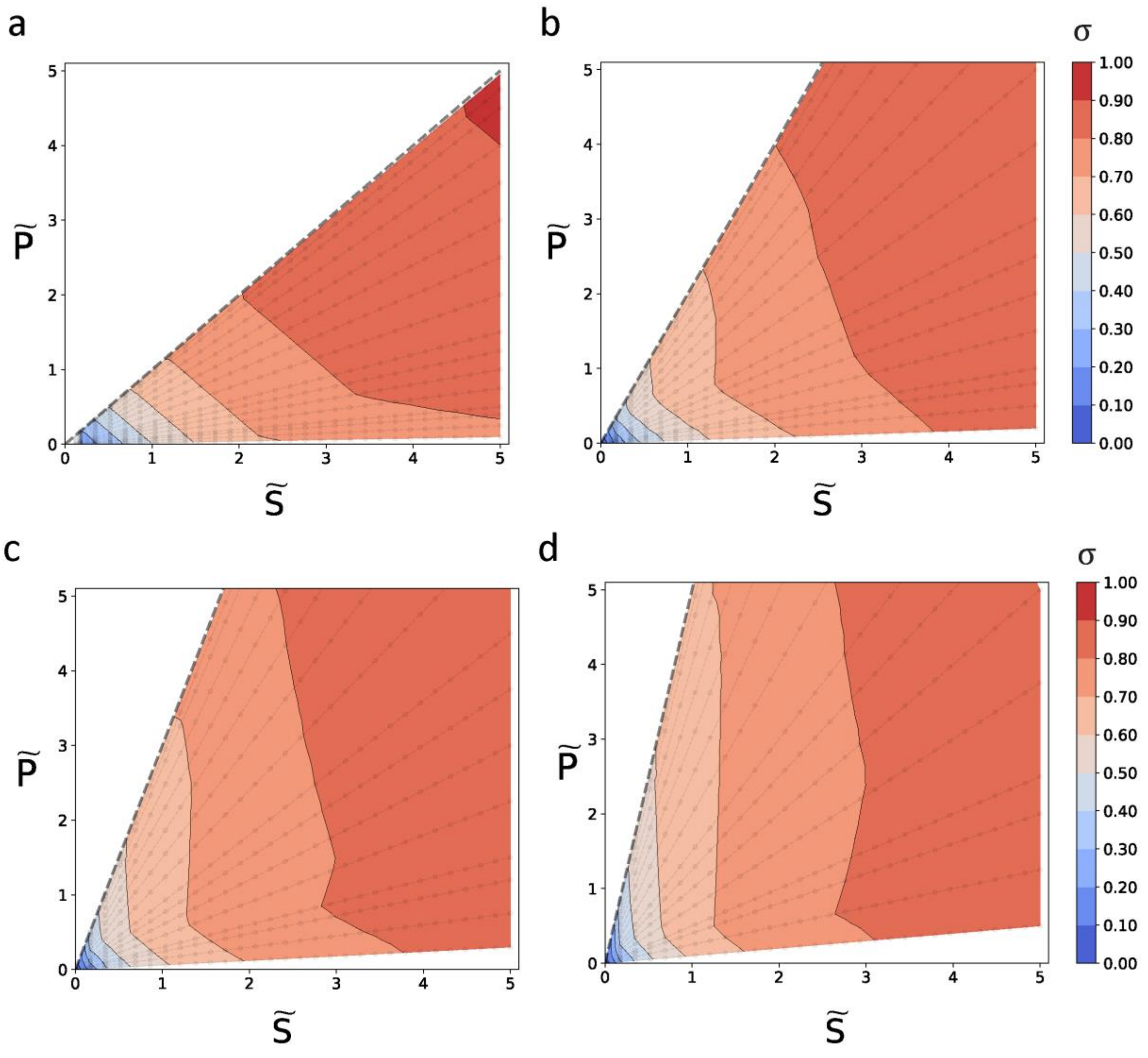

Supplementary Fig. 4: Saturation ($\sigma = 1 - [\widetilde{\mathrm{E}}]$) in the optimal states for different equilibrium constants for the reversible Michaelis-Menten mechanism. a) $\widetilde{K}_{eq} = 1, \Delta \mathrm{G}'^{\circ} = 0 \frac{\mathrm{kcal}}{mol}$ b) $\widetilde{K}_{eq} = 2, \Delta \mathrm{G}'^{\circ} = -0.41 \frac{\mathrm{kcal}}{mol}$ c) $\widetilde{K}_{eq} = 3$, $\Delta \mathrm{G}'^{\circ} = -0.65 \frac{\mathrm{kcal}}{mol}$ d) $\widetilde{K}_{eq} = 5$, $\Delta \mathrm{G}'^{\circ} = -0.95 \frac{\mathrm{kcal}}{mol}$. Dashed line represents the equilibrium line where $\tilde{P} = \widetilde{K}_{eq}\tilde{S}$. Source data are provided as a Source Data file.

Multi-substrate enzyme mechanism

We studied reversible Bi-Uni enzyme mechanism, where substrates might bind to the enzyme in an ordered manner, or in a random-manner (where any substrate can bind first to the enzyme). Our results for the ordered Bi-Uni mechanism show the sensitivity of the net steady-state flux over the

concentration space and captures distinct kinetic designs in accordance with the previous theoretical study[6].

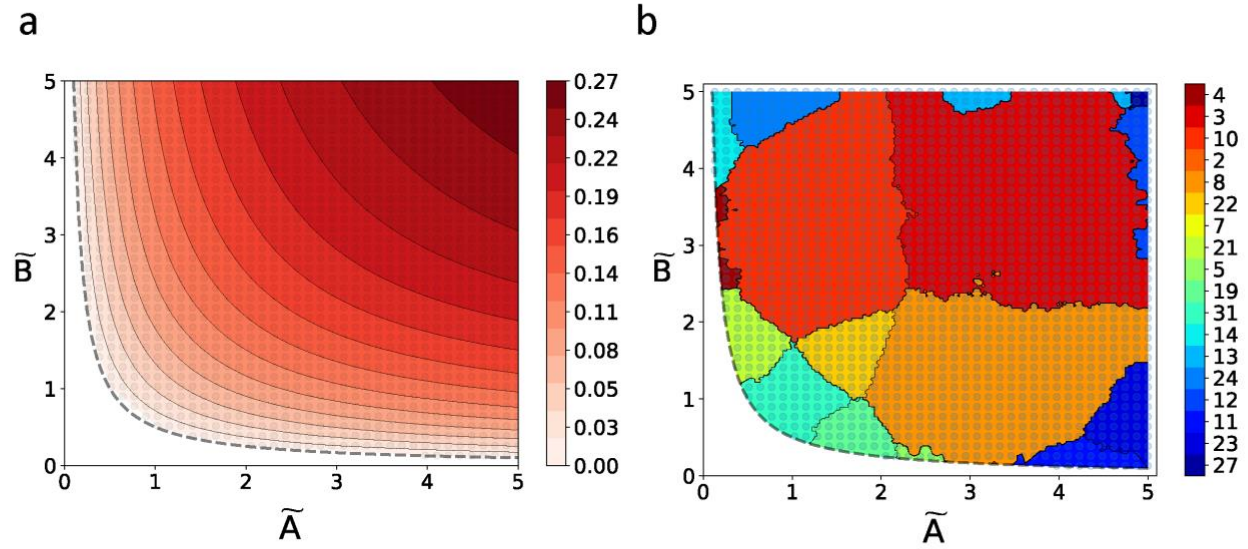

Supplementary Fig. 5: The net steady-state flux ($\tilde{v}_{net}$) and the regions of different kinetic designs at the optimal state for the ordered Bi-Uni mechanism (reproduced from previous theoretical studies[6]). a) Contour plot of the net steady-state flux ($\tilde{v}_{net}$) at optimal state for the ordered Bi-Uni mechanism (Eq. (2) in main text) , b) Contour plot for the regions based on different kinetic designs (defined with respect to the elementary rate constants at their submaximal values, see Supplementary Table 2) reproducing the regions from previous theoretical studies, colors indicate different kinetic designs, numerated according to their original derivation[6] . Scatter points represent sampled points, data is shown for $\widetilde{K}_{eq} = 2$ and $\tilde{P} = 1$. The boundaries of regions are generated using the k-nearest neighbor classifier (k=11). Dashed line represents the equilibrium line where $\tilde{P} = \widetilde{K}_{eq}\tilde{A}\tilde{B}$. Source data are provided as a Source Data file.

Supplementary Table 2: Optimal solution types for the elementary rate constants for the ordered Bi-Uni mechanism. Indicated rate constants take submaximal values for the given kinetic design, whereas the remaining rate constants are at their maximal values (See Supplementary Fig. 5b)

Label	Submaximal rate constants
2	$k_{2,b}$
3	$k_{3,b}$
4	$k_{4,b}$
5	$k_{1,b}, k_{2,b}$
7	$k_{1,b}, k_{4,b}$

8	$k_{2,b}, k_{3,b}$
10	$k_{3,b}, k_{4,b}$
11	$k_{1,f}, k_{2,b}$
12	$k_{1,f}, k_{3,b}$
13	$k_{2,f}, k_{3,b}$
14	$k_{2,f}, k_{4,b}$
19	$k_{1,b}, k_{2,b}, k_{3,b}$
21	$k_{1,b}, k_{3,b}, k_{4,b}$
22	$k_{2,b}, k_{3,b}, k_{4,b}$
23	$k_{1,f}, k_{2,b}, k_{3,b}$
24	$k_{2,f}, k_{3,b}, k_{4,b}$
27	$k_{1,f}, k_{2,f}, k_{3,b}$
31	$k_{1,b}, k_{2,b}, k_{3,b}, k_{4,b}$

151

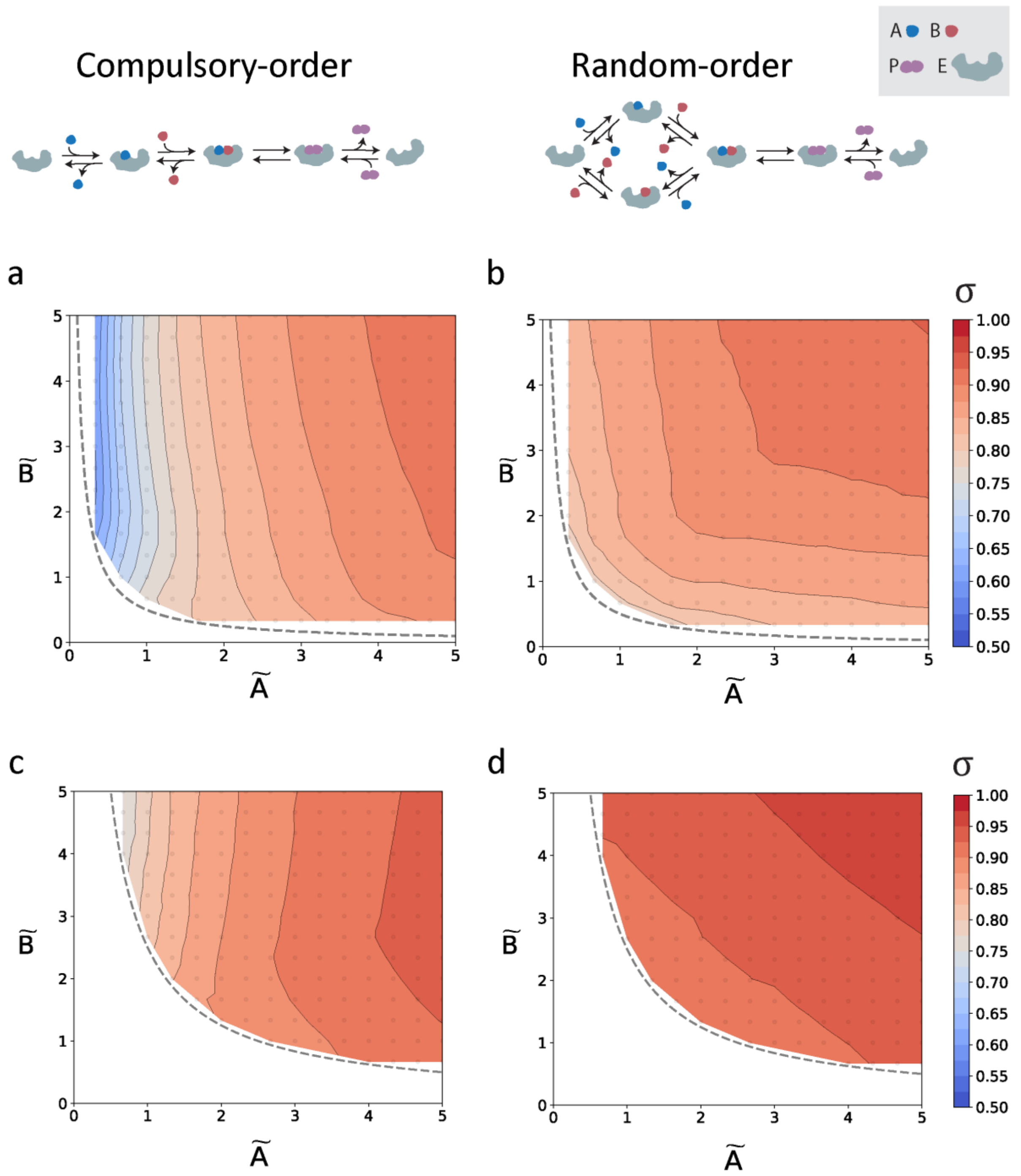

Supplementary Fig. 6: Contour plots for saturation ($\sigma = 1 - [\tilde{\mathrm{E}}]$) in the optimal states for the compulsory-ordered (right) and general (random-ordered) Bi-Uni mechanism (left). for $\widetilde{K}_{eq} = 2$. $\tilde{P} = 1$ for a and b . $\tilde{P} = 5$ for c and d. Dashed line represents the equilibrium line where $\tilde{P} = \widetilde{K}_{eq}\tilde{A}\tilde{B}$. Source data are provided as a Source Data file.

References

1. Noor, E. *et al.* The Protein Cost of Metabolic Fluxes: Prediction from Enzymatic Rate Laws and Cost Minimization. *PLoS Comput. Biol.* **12**, 1–29 (2016).

2. Tepper, N. *et al.* Steady-State Metabolite Concentrations Reflect a Balance between Maximizing Enzyme Efficiency and Minimizing Total Metabolite Load. *PLoS One* **8**, 1–13 (2013).

3. Flamholz, A., Noor, E., Bar-Even, A., Liebermeister, W. & Milo, R. Glycolytic strategy as a tradeoff between energy yield and protein cost. *Proc. Natl. Acad. Sci. U. S. A.* **110**, 10039–10044 (2013).

4. Dourado, H., Mori, M., Hwa, T. & Lercher, M. J. On the optimality of the enzyme–substrate relationship in bacteria. *PLOS Biol.* **19**, e3001416 (2021).

5. Klipp, E. & Heinrich, R. Evolutionary optimization of enzyme kinetic parameters; effect of constraints. *J. Theor. Biol.* **171**, 309–323 (1994).

6. Wilhelm, T., Hoffmann-Klipp, E. & Heinrich, R. An evolutionary approach to enzyme kinetics: Optimization of ordered mechanisms. *Bull. Math. Biol.* **56**, 65–106 (1994).

7. Heckmann, D., Zielinski, D. C. & Palsson, B. O. Modeling genome-wide enzyme evolution predicts strong epistasis underlying catalytic turnover rates. *Nat. Commun.* **9**, 1–9 (2018).

8. Bar-Even, A. *et al.* The moderately efficient enzyme: Evolutionary and physicochemical trends shaping enzyme parameters. *Biochemistry* **50**, 4402–4410 (2011).

9. Labourel, F. & Rajon, E. Resource Uptake and the Evolution of Moderately Efficient Enzymes. *Mol. Biol. Evol.* **38**, 3938–3952 (2021).

10. Segel, I. H. *Enzyme Kinetics: Behavior and Analysis of Rapid Equilibrium and Steady-State Enzyme Systems*. (1975).

11. Heinrich, R. & Hoffmann-Klipp, E. Kinetic parameters of Enzymatic Reactions in States of Maximal Activity; An Evolutionary Approach. *J. Theor. Biol.* **151**, 249–283 (1991).
