# Supplementary material for: Optimal enzyme utilization suggests that concentrations and thermodynamics determine binding mechanisms and enzyme saturations
Source: Nat Commun. 2023 May 5;14:2618. doi: 10.1038/s41467-023-38159-4 (PMC10162984; doi:10.1038/s41467-023-38159-4)
Supplement: Supplementary file 3 — Reporting Summary [file 41467_2023_38159_MOESM3_ESM.pdf]

nature portfolio

Corresponding author(s): Vassily Hatzimanikatis
Last updated by author(s): 05/04/2023

Reporting Summary

Nature Portfolio wishes to improve the reproducibility of the work that we publish. This form provides structure for consistency and transparency in reporting. For further information on Nature Portfolio policies, see our Editorial Policies and the Editorial Policy Checklist.

Statistics

For all statistical analyses, confirm that the following items are present in the figure legend, table legend, main text, or Methods section.

n/a	Confirmed	
☒	☐	The exact sample size (n) for each experimental group/condition, given as a discrete number and unit of measurement
☒	☐	A statement on whether measurements were taken from distinct samples or whether the same sample was measured repeatedly
☒	☐	The statistical test(s) used AND whether they are one- or two-sided<br>*Only common tests should be described solely by name; describe more complex techniques in the Methods section.*
☒	☐	A description of all covariates tested
☒	☐	A description of any assumptions or corrections, such as tests of normality and adjustment for multiple comparisons
☒	☐	A full description of the statistical parameters including central tendency (e.g. means) or other basic estimates (e.g. regression coefficient) AND variation (e.g. standard deviation) or associated estimates of uncertainty (e.g. confidence intervals)
☒	☐	For null hypothesis testing, the test statistic (e.g. F, t, r) with confidence intervals, effect sizes, degrees of freedom and P value noted<br>*Give P values as exact values whenever suitable.*
☒	☐	For Bayesian analysis, information on the choice of priors and Markov chain Monte Carlo settings
☒	☐	For hierarchical and complex designs, identification of the appropriate level for tests and full reporting of outcomes
☒	☐	Estimates of effect sizes (e.g. Cohen's d, Pearson's r), indicating how they were calculated

Our web collection on statistics for biologists contains articles on many of the points above.

Software and code

Policy information about availability of computer code

Data collection	Custom code is written using python 3.6 and run in Docker (20.10.6) containers. Tested with solvers cplex and gurobi with gurobipy 9.1.2 (default implemented on cplex_interface). The main packages used include cplex solver 12.8.0 , optlang 1.5.2, cobrapy v0.17.1 (for sampling). ACHRSampler and OptGpSampler are adapted from their implementation in the cobrapy package v0.17.1. The code is freely available at https://github.com/EPFL-LCSB/open. Other open source packages used in the analysis are also stated in the GitHub repository and in the requirements.txt file in the repository.
Data analysis	Data analysis is done using python 3.6 with open source packages (pandas v1.1.5, numpy v1.19.5, matplotlib v3.3.4, sklearn v0.0). The scripts to generate the results and the figures in the manuscript are available on our GitHub repository.

For manuscripts utilizing custom algorithms or software that are central to the research but not yet described in published literature, software must be made available to editors and reviewers. We strongly encourage code deposition in a community repository (e.g. GitHub). See the Nature Portfolio guidelines for submitting code & software for further information.

Data

Policy information about availability of data

All manuscripts must include a data availability statement. This statement should provide the following information, where applicable:

- Accession codes, unique identifiers, or web links for publicly available datasets
- A description of any restrictions on data availability
- For clinical datasets or third party data, please ensure that the statement adheres to our policy

The scaled concentration ranges for the substrate and product concentrations are adapted from previous theoretical studies for which the references are provided in the main text (Wilhelm et al., 1994). The data generated in this study can be found in the GitHub repository. Source data for the figures generated in this study are provided as a Source Data File.

Human research participants

Policy information about studies involving human research participants and Sex and Gender in Research.

Reporting on sex and gender	No human research participants were involved in our study.
Population characteristics	No human research participants were involved in our study.
Recruitment	No human research participants were involved in our study.
Ethics oversight	No human research participants were involved in our study.

Note that full information on the approval of the study protocol must also be provided in the manuscript.

Field-specific reporting

Please select the one below that is the best fit for your research. If you are not sure, read the appropriate sections before making your selection.

☒ Life sciences ☐ Behavioural & social sciences ☐ Ecological, evolutionary & environmental sciences

For a reference copy of the document with all sections, see nature.com/documents/nr-reporting-summary-flat.pdf

Life sciences study design

All studies must disclose on these points even when the disclosure is negative.

Sample size	No samples were taken in this study.
Data exclusions	No data was excluded from the study.
Replication	All data generated with our computational framework is reproducible as MILP optimal values are a global optimum and are mathematically guaranteed to be replicated. The parameters used for the solver (tolerance, integrality, optimality) and the resolution chosen for discretization is stated explicitly in the main text and/or inside the code.
Randomization	There is no step in the formulation where randomization can be used.
Blinding	There is no step in the formulation where blinding can be used.

Reporting for specific materials, systems and methods

We require information from authors about some types of materials, experimental systems and methods used in many studies. Here, indicate whether each material, system or method listed is relevant to your study. If you are not sure if a list item applies to your research, read the appropriate section before selecting a response.

Materials & experimental systems

n/a	Involved in the study
☒	☐ Antibodies
☒	☐ Eukaryotic cell lines
☒	☐ Palaeontology and archaeology
☒	☐ Animals and other organisms
☒	☐ Clinical data
☒	☐ Dual use research of concern

Methods

n/a	Involved in the study
☒	☐ ChIP-seq
☒	☐ Flow cytometry
☒	☐ MRI-based neuroimaging
